# Impact of a six-week training program on physical fitness and performance of young tennis players: a cluster analysis approach

**DOI:** 10.3389/fspor.2025.1571019

**Published:** 2025-06-06

**Authors:** João P. Oliveira, Daniel A. Marinho, Tatiana Sampaio, Bulent Kilit, Jorge E. Morais

**Affiliations:** ^1^ Department of Sport Sciences, University of Beira Interior, Covilhã, Portugal; ^2^ Research Center in Sports Sciences, Health Sciences and Human Development (CIDESD), Covilhã, Portugal; ^3^ Research Centre for Active Living and Wellbeing (LiveWell), Instituto Politécnico de Bragança, Bragança, Portugal; ^4^ Faculty of Sport Sciences, Tokat Gaziosmanpasa University, Tokat, Türkiye; ^5^ Department of Sport Sciences, Instituto Politécnico de Bragança, Bragança, Portugal

**Keywords:** youth tennis, cluster analysis, physical fitness, intervention, performance

## Abstract

This study aimed to classify young tennis players into sub-groups (clusters) based on their performance and physical fitness determinants and to analyze their cluster assignment change over six weeks of intervention. A sample of young athletes aged 10–14 years was used, and players were classified according to their International Tennis Number (ITN) and various physical fitness metrics after a six-week specific training program. Clusters were also analyzed for cluster assignment changes across pre-test and post-test interventions. The results showed that the performance variables in all clusters improved at the end of the intervention. Specifically, ITN scores improved by an average of 15% (*p* < 0.05), the 5-m sprint times improved by 8.5% on average (*p* < 0.01), and the T-drill agility test showed a 10% reduction in completion time (*p* < 0.01). However, key determinants for cluster formation assessment scores differed, meaning that the training resulted in the physical fitness profiles changing over time. Improvement in 5-m sprint and T-drill agility tests showed that the current program was effective in improving movement characteristics that are vital to a tennis player. Movement between clusters was observed, with some players improving their classification while others regressed, highlighting the need for individualized training interventions to optimize player development. Individualized responses to training are common among young athletes, reflecting varying developmental stages. Clustering can help tailor training programs to the specific needs of different groups. In summary, this study highlights the effects of specific training on young tennis players and emphasizes the necessity of considering individual differences in growth and training responses.

## Introduction

Identifying and developing sports talent is fundamental to optimize athletic performance (1). Agility and physical performance have become very important in sports requiring spontaneity of decisions and movement accuracy (2). Tennis is characterized by physical and technical demands, where players must master a combination of skills that enable them to respond quickly and accurately during matches (3). This involves the necessity to change directions quickly and effectively. Change of direction (COD) ability, as defined by Jones et al. (4), refers to “the ability to decelerate, reverse, or change movement direction and accelerate again”. Agility tests, such as the T-drill, which is based on the analysis of movement patterns like sprint, lateral shuffle, and backpedal (5), are widely used for measuring this ability in sports (6–8). Such tests are crucial for setting standards and for categorizing athletes based on performance levels (9).

While agility and COD are undoubtedly critical, performance in tennis also relies on other physical attributes, including speed, strength, balance, and aerobic capacity (10). These components collectively contribute to a player's ability to sustain high-intensity actions, endure long rallies, and maintain peak performance over extended matches. To capture this complexity, performance assessments often include a range of tests, such as linear sprints to evaluate acceleration, repeated sprint ability (RSA) for short-term endurance, and VO_2max_ measurements to gauge aerobic capacity (5, 10). Integrating these assessments allows for a comprehensive profile of an athlete's physical readiness, providing coaches with valuable insights for individualizing training programs based on specific strengths and weaknesses (9).

The International Tennis Number (ITN) is a widely used index to classify players based on their overall tennis proficiency, particularly technical and tactical abilities. It includes assessments of groundstroke depth, serve accuracy, and court mobility, all reflecting critical skills for effective tennis performance (11). However, the ITN does not directly assess physical components such as short-distance acceleration, aerobic fitness, or change of direction ability—attributes that are also critical for tennis performance, particularly among developing players. Therefore, physical performance tests like the T-drill (12), 20 m sprint (13), and repeated sprint ability (14) have been recommended as complementary assessments to provide a more complete profile of a player's capabilities.

Grouping athletes based on performance characteristics helps coaches identify specific group needs (15). This approach allows for targeted training intensity, optimized recovery protocols, and strategic adjustments to prevent injury (16), ensuring that each athlete develops essential skills safely and effectively. Previous studies have explored clustering methods to categorize athletes based on performance-related characteristics, providing valuable insights into training adaptation and talent development. For example, in a previous work (17), it was demonstrated that clustering was effective in distinguishing young swimmers’ performance levels using biomechanical and physiological variables, highlighting that athletes in higher-performance clusters often share distinct physical and technical traits. Similarly, Sampaio et al. (16) showed how clustering young football players based on lower limb strength, power, dynamic balance, speed, and change of direction revealed group-specific physical profiles, suggesting tailored training programs for each cluster. Additionally, research by Rein et al. (15) supports clustering in sports, indicating that these methods can reveal underlying patterns in athletes’ strengths and limitations, offering a framework for individualized training. In agility-based sports, agility metrics and change-of-direction speed are critical differentiators across performance levels (2).

Clustering techniques have been extensively applied across various sports, including soccer (16), swimming (18), and basketball (19), to classify athletes based on performance metrics. These studies have demonstrated that clustering can uncover distinct physical and technical profiles, enabling tailored training approaches for elite or mature athletes. In tennis, clustering applications have primarily focused on older (20, 21) or professional (22) players, leaving a noteworthy gap in understanding how these methods might be used for youth athletes, particularly at the early stages of skill development. For younger athletes, individualized training strategies informed by clustering could be especially beneficial in guiding foundational skill progression. This study aims to address this gap by focusing on young tennis players, providing insights into how clustering can reveal baseline performance differences and capture developmental changes over time in response to training. A recent study demonstrated that combining agility, sprinting, and aerobic capacity effectively predicted ITN levels, suggesting that these measures can serve as indicators of overall tennis performance (5). The clustering approach could contribute to more targeted training protocols and a deeper understanding of progression trajectories in young players, informing new frameworks for youth athlete development.

Therefore, the aim of this study was to: (i) classify young tennis players into sub-groups (clusters), according to their performance (ITN) and a set of physical fitness variables related to tennis based on a specific training program, and; (ii) analyze the individual changes between sub-groups (clusters) of each player between the pre- and post-test. It was hypothesized that the variables responsible for the sub-group formation would differ between moments of assessment and that the players would also change their cluster assignment, i.e., the cluster assignment would be different between moments of assessment.

## Methods

### Research design

All players underwent a six-week on-court training program with three sessions per week, conducted on an indoor hard court. Each session followed a standardized structure: (i) 10-min warm-up (light jogging, dynamic and passive stretching, tennis-specific movements), (ii) 20-min tennis-specific drills (e.g., serve-return, groundstroke combinations, volley execution, and placement), and (iii) 20–40-min on-court conditioning designed to enhance acceleration, stroke quality under fatigue, and transition movements. These included forehand–backhand transition drills performed at maximal/submaximal intensities, with racquet and ball use. Work sets were organized into 2–3 sets of 5–6 repetitions (30–60 s work, 30–60 s rest), supervised by experienced coaches. Drills were designed to hit target zones on the court, and were delivered with a consistent ball feed rate (∼1 ball every 3 s). Each session ended with 8–20 min of high-intensity interval running (above 85% HRmax) and a 10-min cooldown. Heart rate was monitored to ensure appropriate training load (5).

### Participants

Twenty young male tennis players (age: 13.6 ± 0.2 years; body mass: 51.9 ± 8.4 kg; height: 161.3 ± 8.3 cm) classified as Tier 3 athletes (23) were evaluated. To be included in the study, all players should be fit and competitively active, free of injury, and training periodically over the past six months before data collection. Detailed procedures about the study protocol were provided to players and their parents, and voluntary written consent was obtained. The study was carried out following the principles of the Helsinki Declaration and approved by the Local University Research Ethics Committee (47940-01).

### International tennis number

The ITN (in arbitrary units—a.u) test was performed to assess the player's technical skills and considered as the performance indicator. That is, players with the greatest ITN were considered the best performers. This test consists of 5 technical elements: (i) groundstroke depth; (ii) groundstroke accuracy; (iii) volley depth; (iv) serve, and; (v) mobility. Detailed information about the test protocol can be found here (11).

### Maximal oxygen uptake (Vo_2max_)

The maximal oxygen uptake (VO_2_max, ml/kg/min) was estimated using the Hit and Turn Tennis (HTT) test, a field-based aerobic assessment specific to tennis (24). Heart rate was continuously monitored during the test using Polar V800 devices (Polar Inc., Finland), not as a component of the estimation formula, but rather as a safety measure to allow evaluators to monitor effort levels and ensure proper execution of the test. The VO_2_max value was calculated using the following equation: VO_2_max = 33.0 + (1.66 × HTT), where HTT corresponds to the final stage completed by the player (a.u) (24).

### T-drill

The T-drill agility test (in s) assessed the players’ agility performance. This test consisted of sprinting from a standing point to a cone placed 9.14-m away before side-shuffling to their left without crossing their feet to another cone placed 4.57-m away. After reaching this last cone, they shuffle right to a third cone placed 9.14-m away, side-shuffle back to the middle cone, and run back to the starting point. This test performance is based on time, where shortest times are related to better performances. A wireless photocell system (Witty, Microgate, Bolzano, Italy) was used to measure time (16). The photocells were placed in the first cone which corresponded to the start and finish of the test. Each player performed the test two times with a 10-min rest between them. The fastest one was used for analysis.

### Sprint test

Players performed a linear 20-m sprint test. The wireless photocell system (Witty, Microgate, Bolzano, Italy) was used to measure time with four pairs of photocells positioned at the 0 m (start), 5 m, 10 m, and 20 m marks. This setup enabled accurate collection of split times for the 5-, 10-, and 20-m segments. Players were instructed to perform maximally until they reached the 20-m timing gate. The players performed two trials with a 10-min rest between them. The fastest one was used for analysis.

### Repeated sprint ability (RSA)

The RSA test (in s) consisted of six repetitions of maximal 2 × 15-m shuttle sprints with approximately 14-s recovery between sprints (14). An evaluator gave the start. The same photocell system (Witty, Microgate, Bolzano, Italy) was used to measure time by using only one pair of photocells (the same for start and finish). The players performed two trials with a 10-min rest between them and the fastest one was used for analysis. Afterwards, the mean of the six repetitions (in s) was used as a performance indicator (14).

### Statistical analysis

The data distribution was assessed using the Shapiro–Wilk test, revealing a normal distribution. Mean ± standard deviations were calculated as descriptive statistics. Cluster modeling was performed using a non-hierarchical approach, i.e., k-means approach (15). Despite this approach allowing an à priori definition of several clusters to be used in advance, the elbow method was used to understand the number of clusters to be retained for analysis. Standardized *z*-scores were used to ensure a coherent comparison of data sets with different magnitudes and/or units. The one-way ANOVA was used to identify the main determinants responsible for establishing the clusters (*p* < 0.05), and the eta square (*η*^2^) was selected as effect size index: (i) without effect if 0 < *η*^2^ < 0.04; (ii) minimum if 0.04 < *η*^2^ < 0.25; (iii) moderate if 0.25 < *η*^2^ < 0.64 and; (iv) strong if *η*^2^ > 0.64 (25). Discriminant analysis, based on the stepwise method, was used to validate the cluster formation.

## Results

Table 1 presents the descriptive data per cluster and for the pre- and post-test. In both the pre- and post-test, Cluster 1 included the best performers for the ITN, cluster 2 the intermediate, and cluster 3 the poorest. In the pre-test, the 20-m sprint was the variable that better discriminated the cluster formation (*F* = 16.18, *p* < 0.001, *η*^2^ = 0.66). In the post-test, it was the 5-m sprint (*F* = 18.93, *p* < 0.001, *η*^2^ = 0.69). In the pre-test, cluster 1 was characterized by a high VO_2max_, great ITN, and fast 10-m sprints (lower time indicates better performances). Cluster 2 by fast 5-m sprints (lower time indicates better performances), slow 20-m sprints (poorer performances), and low VO_2max_. Cluster 3 by a slow T-drill (more time indicates poor performances), small ITN, and slow 5-m sprints (more time indicates poor performances). In the post-test, cluster 1 was characterized by a great ITN, fast T-drill (less time indicates best performances) and slow 10-m sprints (more time indicates poor performances). Cluster 2 by fast 5-m and 10-m sprints (less time indicates best performances), and fast RSA (less time indicates best performances). Cluster 3 by slow T-drill, RSA, and 5-m sprints (more time indicates poor performances).

**Table 1:** Descriptive statistics of all variables measured per cluster in the pre- and post-test. The F-ratios showing the determinant factors responsible for the cluster formation (and the effect size—*η*^2^) are also presented.

	Pre-test							
	Cluster 1 (*N* = 6)		Cluster 2 (*N* = 9)		Cluster 3 (*N* = 5)		F-ratio (p)	*η* ^2^
	Mean ± SD	*z*-score	Mean ± SD	*z*-score	Mean ± SD	*z*-score		
ITN [a.u.]	231.17 ± 18.44	0.6335	220.11 ± 23.37	0.1747	190.00 ± 3.53	−1.0747	6.82 (0.007)	0.45
VO_2max_ [ml/kg/min]	47.33 ± 0.75	0.9809	43.94 ± 1.50	−0.6743	45.40 ± 2.07	0.0366	9.20 (0.002)	0.52
5-m sprint [s]	1.13 ± 0.04	−0.3440	1.13 ± 0.04	−0.3440	1.19 ± 0.02	1.0321	5.07 (0.019)	0.37
10-m sprint [s]	2.10 ± 0.07	−0.6062	2.17 ± 0.10	0.0783	2.22 ± 0.08	0.5863	2.25 (0.136)	0.21
20-m sprint [s]	3.52 ± 0.10	−0.4649	3.79 ± 0.07	0.8479	3.41 ± 0.21	−0.9682	16.18 (<0.001)	0.66
RSA [s]	6.58 ± 0.12	0.0616	6.55 ± 0.10	−0.1639	6.60 ± 0.11	0.2211	0.23 (0.794)	0.03
T-drill [s]	12.48 ± 0.11	−0.5358	12.52 ± 0.14	−0.2954	12.76 ± 0.05	1.1747	8.33 (0.003)	0.50
	Post-test							
	Cluster 1 (*N* = 5)		Cluster 2 (*N* = 9)		Cluster 3 (*N* = 6)		F-ratio (p)	*η* ^2^
	Mean ± SD	*z*-score	Mean ± SD	*z*-score	Mean ± SD	*z*-score		
ITN [a.u.]	252.80 ± 9.44	0.8207	234.11 ± 21.26	−0.0314	220.83 ± 21.77	−0.6368	3.74 (0.045)	0.31
VO_2max_ [ml/kg/min]	48.80 ± 2.16	0.0776	49.17 ± 2.06	0.2404	47.67 ± 2.65	−0.4253	0.80 (0.465)	0.09
5-m sprint [s]	1.10 ± 0.04	0.5117	1.03 ± 0.02	−0.8813	1.13 ± 0.02	0.8955	18.93 (<0.001)	0.69
10-m sprint [s]	2.14 ± 008	0.8728	2.02 ± 0.04	−0.5025	2.07 ± 0.09	0.0264	4.01 (0.038)	0.32
20-m sprint [s]	3.48 ± 0.25	0.1354	3.45 ± 0.19	−0.0150	3.43 ± 0.21	−0.0902	0.06 (0.938)	0.01
RSA [s]	6.40 ± 0.16	0.1888	6.26 ± 0.09	−0.7183	6.51 ± 0.08	0.9201	9.25 (0.002)	0.52
T-drill [s]	12.16 ± 0.15	−0.4974	12.16 ± 0.13	−0.4834	12.49 ± 0.14	1.1397	12.03 (0.001)	0.59

ITN, international tennis number; VO_2max_, maximal oxygen uptake; RSA, repeated sprint ability; *η*^2^, eta squared (effect size index).

**Figure 1:**
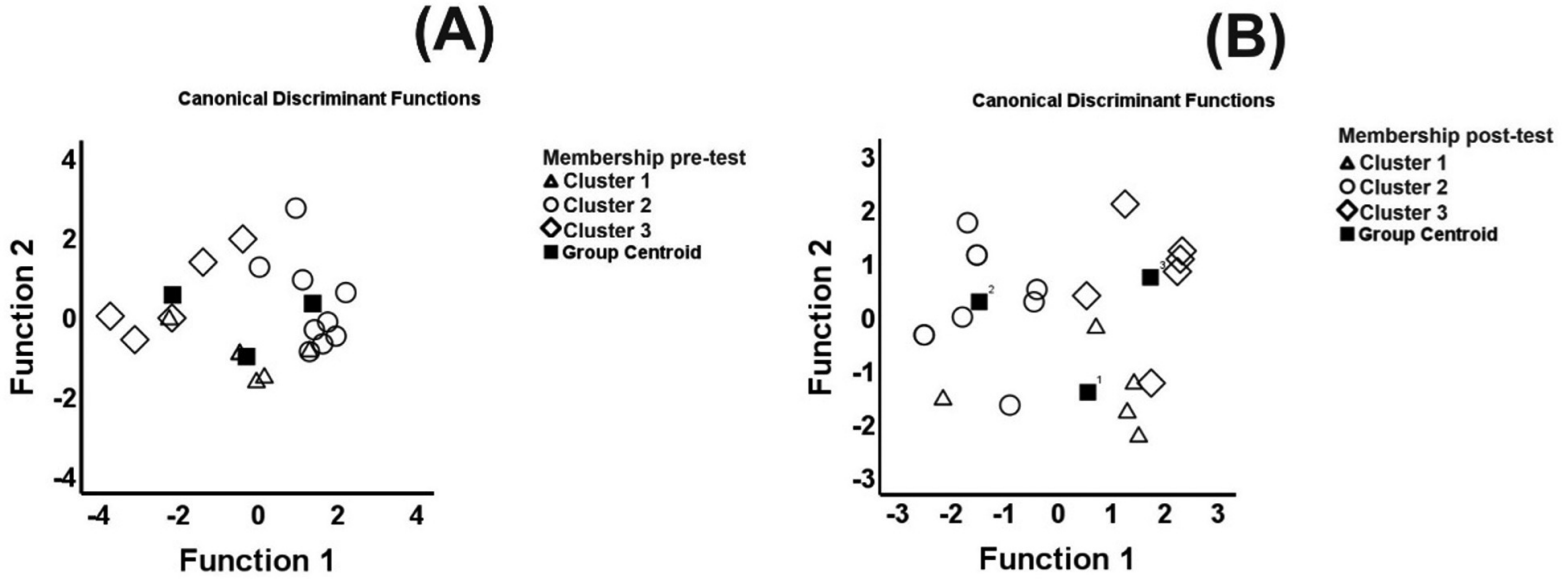
Territorial map in each moment of assessment [**(A)** pre-test; **(B)** post-test].

Figure 1 depicts the stepwise discriminant analysis as qualitative assessment for the pre- (panel A) and post-test (panel B). Based on the results of the territorial map, the discriminant analysis showed good compactness/separation with a correct classification of the original groups (pre-test: 90.0%; post-test: 85.0%) and a correct classification of cross-validated groups (pre-test: 85.0%; post-test: 80.0%).

In each evaluation moment, two functions were extracted. In the pre-test, function 1 was mainly defined by the 20-m sprints explaining 82.6% of the variance (Λ = 0.196, *X*^2^ = 26.891, *p* < 0.001). Function 2 was mainly defined by the T-drill explaining 17.4% of the variance (Λ = 0.665, *X*^2^ = 6.725, *p* = 0.010). The classification functions are as follows:

(1) Cluster1=203.20⋅20m+844.06⋅Tdrill−5,626.56

(2) Cluster2=219.53⋅20m+846.55⋅Tdrill−5,717.32

(3) Cluster1=196.79⋅20m+862.86⋅Tdrill−5,841.69

In the post-test, function 1 was mainly defined by the 5-m sprint explaining 73.8% of the variance (Λ = 0.166, *X*^2^ = 29.602, *p* < 0.001). Function 2 was mainly defined by the T-drill explaining 26.2% of the variance (Λ = 0.550, *X*^2^ = 9.879, *p* = 0.002). The classification functions are as follows:

(4) Cluster1=−149.31⋅5m+633.92⋅Tdrill−3,771.64

(5) Cluster2=−240.83⋅5m+643.32⋅Tdrill−3,788.35

(6) Cluster3=−164.98⋅5m+652.68⋅Tdrill−3,985.35

**Figure 2:**
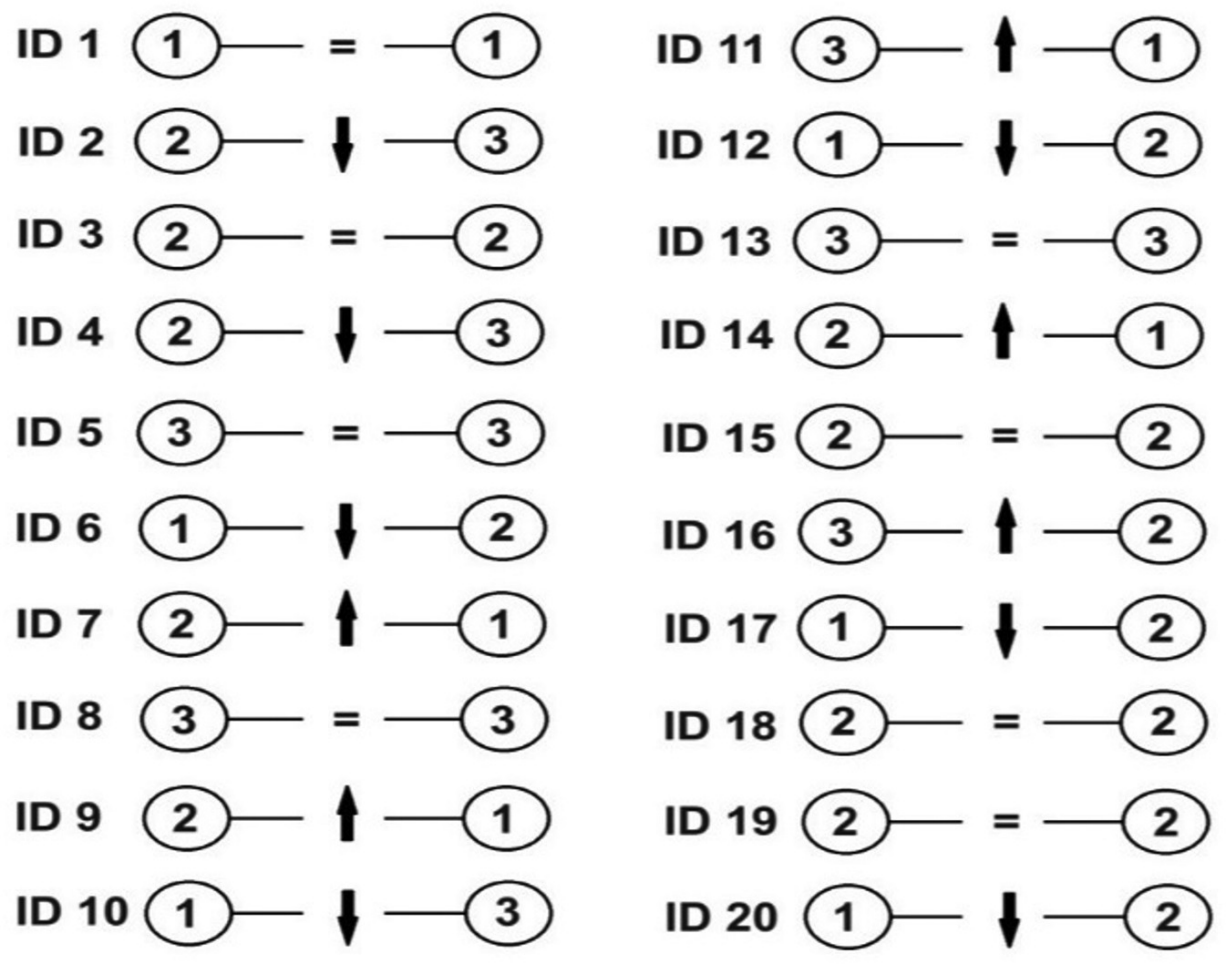
Individual changes between moments of assessment. ID #—corresponds to the player identification; ellipsis—corresponds to the cluster; equal signal—corresponds to cluster maintenance; up arrow—corresponds to a cluster shift, i.e., to a better performer cluster; down arrow—corresponds to a cluster shift, i.e., to a poorer performer cluster.

Figure 2 depicts the players’ cluster assignment change between the pre- and post-test. Of the 20 athletes, 8 maintained their cluster assignment (one in Cluster 1, four in Cluster 2, and three in Cluster 3). Seven athletes decreased their cluster assignment, i.e., changed to a poorer cluster. Five athletes increased their cluster assignment, i.e., changed to a better cluster.

## Discussion

The aim of this study was to: (i) classify young tennis players into sub-groups (clusters), according to their performance (ITN) and a set of physical fitness variables related to tennis based on a specific training program, and; (ii) analyze the individual changes between sub-groups (clusters) of each player between the pre- and post-test. The International Tennis Number (ITN) is a widely recognized rating system used to assess the skill level of tennis players, ranging from beginner to advanced. In this study, ITN was used as the main indicator to classify players into performance-based clusters. The clusters were labeled as best, intermediate, and lower performers based on ITN scores, providing a clear distinction of performance levels among the players. The main findings indicate that, overall, the selected variables improved between the pre- and the post-test in all three clusters. It was found that, at each moment, the variables responsible for the characterization of each cluster were different, and some players shifted between clusters. These results provide insights into the effects of targeted training on young athletes and underscore the variability in physical development during adolescence, highlighting the importance of individualized approaches to maximize each player's potential.

The determinants of the clusters at baseline were different from those after the 6-week intervention. Specifically, in baseline, the 20-m sprint time was a key determinant for distinguishing between clusters, along with agility metrics. This suggests that, initially, speed over longer distances and agility were primary indicators of performance level. After the intervention, however, the 5-m sprint emerged as the critical factor, reflecting a shift towards the importance of initial acceleration as players adapted to the training program. This change in determinants highlights how the training intervention led to different adaptations in physical profiles, emphasizing the dynamic nature of physical fitness development. These findings align with earlier research underscoring the significance of speed and agility during the early stages of talent development (2, 9).

The change in cluster assignment between the pre- and post-test emerged was a noteworthy observation. Not all players initially classified in the best-performing cluster at baseline remained there post-intervention, and similarly, some players moved from lower-performing clusters to higher ones. For instance, the player with ID 10 improved in almost all performance metrics but moved from Cluster 1 (best) at baseline to Cluster 3 (lower) post-intervention. Notably, five athletes improved their classification, moving to a higher-performing cluster, while seven athletes declined. This observation indicates that while most players responded well to the training program, others might require additional or different stimuli to maximize their development, which aligns with studies showing differential adaptation rates among young athletes (17). Given these individualized adaptations, it is crucial to consider the broader implications for training strategies aimed at enhancing specific skills and optimizing overall player development. This variability aligns with findings in other sports, such as swimming (17) and football (16), where individualized responses to training have been reported. The study by Morais et al. (17) on young swimmers revealed that athletes changed clusters over two competitive seasons, suggesting that the factors determining cluster assignment vary significantly over time and that adaptation to training stimuli can be highly individualized. Similarly, in our study, we observed movement between clusters, with some players improving while others regressed. This variability highlights the dynamic nature of physical development during adolescence and supports the idea that individualized training approaches are essential for optimizing player development. Morais et al. (17) also found consistent improvements across all clusters, whereas our findings showed that not all players progressed; some players regressed, indicating that the training stimulus might not have been equally effective for all participants. Moreover, the fact that seven athletes shifted to lower-performing clusters following the intervention warrants further attention. In some cases, athletes showed improvements in absolute performance variables but were still reassigned to lower-performing clusters. This may suggest that their rate of improvement was comparatively smaller than that of their peers, leading to a relative decline in cluster assignment. Additionally, transient factors such as fatigue or underperformance during post-testing may have influenced the outcomes. Finally, the nature of the k-means clustering algorithm, which classifies based on multivariate similarity, means that even minor profile shifts can lead to reclassification, especially in borderline cases. These findings reinforce the need to interpret cluster reassignments not in isolation, but in the context of both absolute improvements and relative positioning within the group. Additionally, the findings of Sampaio et al. (16), also reported that in young football players, individualized training was crucial for improving physical capacities, especially considering the distinct developmental stages of athletes.

The general improvement in performance across clusters following the intervention highlights the effectiveness of the training program in enhancing physical fitness. In particular, the significant improvements in the T-drill agility test, a key contributor to cluster formation, emphasize the relevance of directional change skills in youth tennis. These findings reinforce prior evidence that agility is a critical differentiator in tennis performance (3).

Building on these findings, adapted recommendations can be proposed based on the cluster profiles. For example, players in Cluster 3 (lowest performers) demonstrated weaker results in agility and acceleration (T-drill, 5-m sprint), indicating a need for targeted neuromuscular and movement skill development. Specific drills focusing on deceleration, reacceleration, and multi-directional quickness could be emphasized. In Cluster 2, players had better sprinting profiles but lower ITN scores, suggesting they may benefit from integrating physical training with more advanced technical-tactical drills under fatigued conditions to simulate match play demands. Cluster 1 athletes generally showed balanced profiles but, maintaining or progressing further would require greater emphasis on high-intensity tennis-specific conditioning and complex decision-making scenarios. These differentiated training priorities align with the framework presented by Kolman et al. (26), who showed that high-performing tennis players display not only superior technical and tactical skills but also underlying physical qualities that support decision-making and movement execution. Thus, designing training content based on cluster-specific needs may maximize developmental efficiency. In addition to the physical metrics used, it is important to highlight the contribution of the ITN score as a composite index that encompasses technical and tactical dimensions of tennis performance. As part of the clustering input, the ITN helped differentiate players not only based on fitness capacities, but also based on gameplay effectiveness, stroke control, and tactical consistency. This means that movement between clusters may have been influenced by shifts in both physical preparedness and technical-tactical execution. For instance, a player improving fitness test results but underperforming in match-like situations may still be reassigned to a lower-performing cluster due to ITN changes. Including the ITN score therefore offers a broader view of development and performance beyond isolated physical indicators.

These insights carry practical relevance for coaches aiming to implement individualized training strategies. Clustering athletes based on physical and performance profiles enables training to be tailored more precisely to group-specific needs (15). For instance, lower-performing athletes can focus on improving foundational capacities such as agility and speed, while more advanced players may benefit from refining technical execution and tactical decision-making under match-like conditions. This targeted approach supports optimized development by aligning training interventions with each player's performance profile (16, 17).

While clustering is fundamentally a group-based classification method, it does not contradict the principles of individualization. Instead, it provides a practical framework for segmenting athletes based on shared characteristics, thereby supporting what could be termed group-tailored individualization. This approach allows coaches to design differentiated training interventions that address the common needs of each cluster, while still adapting within those parameters to the individual strengths and weaknesses of each athlete. Thus, clustering serves as a bridge between population-level analysis and individualized coaching, facilitating more nuanced and efficient development strategies.

This study has several limitations that should be acknowledged. First, it involved a relatively small, homogeneous, and single-gender sample, which may limit the generalizability of the findings to broader populations. Second, the absence of a control group restricts internal validity, as improvements observed over time may have been partially influenced by external factors such as biological maturation, seasonal match exposure, or test familiarity. Third, while the clustering approach offered valuable insight into performance-based grouping, it was applied at only two time points, limiting the ability to capture long-term developmental trajectories.

Another important limitation is the absence of biological maturity indicators, such as peak height velocity or maturity offset. Given that youth athletes of the same chronological age may differ significantly in their biological development, this omission may have affected cluster assignment and the interpretation of training responses (27). Furthermore, the study focused primarily on physical performance metrics, which—although relevant—represent only one dimension of tennis performance. In a skill-based sport, technical and tactical aspects play a central role, and future research should seek to integrate more comprehensive skill assessments into performance profiling.

Finally, although clustering is a group-based analytical method, it was used here to support individualized training strategies. This conceptual framework assumes that athletes within a cluster share performance needs, allowing training to be adapted accordingly. However, this form of group-tailored individualization is not a substitute for truly individualized planning, and future studies should continue to explore how machine learning approaches can enhance personalized training in dynamic sport environments. Despite these limitations, the current study contributes important insights into how clustering can inform training design and talent development in youth tennis.

## Conclusion

This study demonstrates that clustering young tennis players based on physical fitness metrics can effectively capture the dynamics of performance changes over a short training period. The observed improvements in ITN scores and key performance metrics support the effectiveness of the six-week training intervention. However, the fact that some players improved while others regressed underscores the importance of considering individual variability in training responses. Future research should further explore individualized training adaptations and consider biological maturation factors to enhance the development of young tennis players.

## Data Availability

The raw data supporting the conclusions of this article will be made available by the authors, without undue reservation.
